# First Report of an *SH2D1A* Mutation Associated with X-Linked Lymphoproliferative Disease in Turkey

**DOI:** 10.4274/tjh.2017.0445

**Published:** 2018-08-05

**Authors:** Selman Kesici, Ebru Yılmaz Keskin, Samuel C.C. Chiang, Çiğdem Seher Kasapkara, Takuya Sekine, Meltem Akçaboy, Ali Fettah, Yenan T. Bryceson

**Affiliations:** 1 Dr. Sami Ulus Maternity and Children’s Health and Diseases Training and Research Hospital, Clinic of Pediatric Intensive Care, Ankara, Turkey; 2 Süleyman Demirel University Faculty of Medicine, Department of Pediatric Hematology and Oncology, Isparta, Turkey; 3 Karolinska University Hospital Huddinge, Karolinska Institute, Center for Hematology and Regenerative Medicine, Department of Medicine, Stockholm, Sweden; 4 Dr. Sami Ulus Maternity and Children’s Health and Diseases Training and Research Hospital, Clinic of Pediatric Metabolism and Nutrition, Ankara, Turkey; 5 Dr. Sami Ulus Maternity and Children’s Health and Diseases Training and Research Hospital, Clinic of Pediatrics, Ankara, Turkey; 6 Dr. Sami Ulus Maternity and Children’s Health and Diseases Training and Research Hospital, Clinic of Pediatric Hematology and Oncology, Ankara, Turkey

**Keywords:** Lymphoproliferative disease, Hemophagocytosis, Epstein-Barr virus

## To the Editor,

X-linked lymphoproliferative disease (XLP) is a rare disorder characterized by an extreme vulnerability to Epstein-Barr virus (EBV) infection, frequently resulting in hemophagocytic lymphohistiocytosis (HLH) [1]. XLP-1, its more common subtype, is caused by defects in the *SH2D1A* gene that encodes the signaling lymphocyte activation molecule-associated protein (SAP), which regulates the activation of T lymphocytes [2], whereas XLP-2 is caused by mutations in the *XIAP*gene, also known as *BIRC4* [3]. 

We present here an XLP-1 patient with a family history of the death of multiple male children, who presented with EBV-triggered fatal HLH. To our knowledge, this is the first report of an *SH2D1A*mutation from Turkey.

**Case:** The 19-month-old male patient, admitted with the complaints of fever and abdominal distention, had pale appearance, fever (body temperature: 39.5 °C), dyspnea, tachycardia, abdominal distention, and hepatosplenomegaly. Laboratory findings are summarized in Table 1.

**Figure 1:**
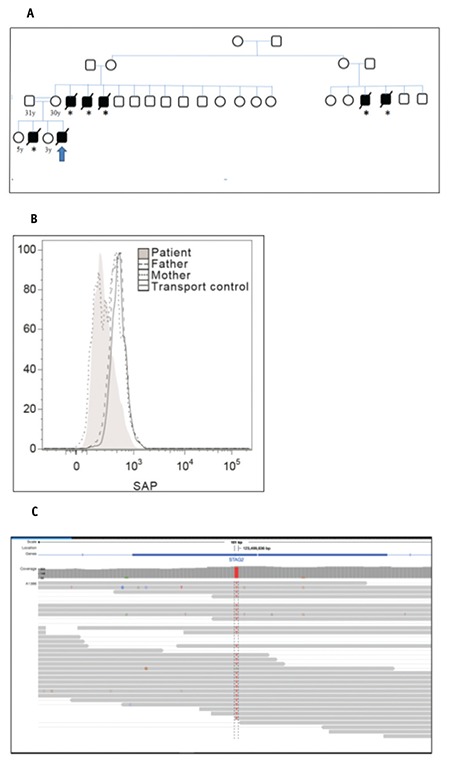
A) Pedigree of the family demonstrating loss of six male children, compatible with X-linked recessive inheritance of disease. *All of the designated deaths occurred between 1 and 3 years of age. The propositus is indicated with an arrow; B) The levels of signaling lymphocyte activation molecule-associated protein (SAP) expression on dim natural killer cells of the patient and the parents by intracellular SAP analysis; C) Identification of the c.163C>T (p.Arg55Ter) mutation in the SH2D1A gene by sequencing analysis in the index case.

In the family history, the death of a 2-year-old male sibling with the clinical diagnosis of HLH and of five young male children of unknown etiology among maternal relatives was noted (Figure 1).

The patient received intravenous immunoglobulin. However, in the follow-up, fever recurred and his general condition worsened. Bone marrow aspiration revealed hemophagocytosis. Therefore, the patient fulfilled the HLH diagnostic criteria. Plasma exchange was performed. Blood products, antimicrobials, and supportive therapeutic agents were used as indicated.

The results of EBV serologic testing and polymerase chain reaction were both reported as positive. On the 6^th^ hospitalization day, the HLH-2004 protocol treatment was initiated, and rituximab therapy was planned. Continuous veno-venous hemodialysis was performed. However, the vital signs of the patient deteriorated further and active gastrointestinal bleeding was observed. The patient died on the 10^th^ day of hospitalization.

In the cytotoxic lymphocyte activity analysis, low SAP expression in addition to signs of severe immunoactivation was detected (Figure 1). In the genetic analysis performed in the Clinical Genetics Unit of Karolinska University Hospital, Stockholm, Sweden, the c.163C>T (p.Arg55Ter) mutation in the *SH2D1A* gene, described previously as pathologic [4], was identified (Figure 1). Genetic counseling was provided to the family. This letter was written after receiving informed consent from the parents.

We report here an XLP-1 case in which the patient presented with EBV-associated HLH. Although no genetic analysis was performed among the male relatives of the patient lost previously in childhood, XLP-1 seems to be the underlying cause in those children as well.

In XLP cases, the most common clinical manifestation is fulminant infectious mononucleosis (frequency: 58%, survival: 4%). Death is generally attributable to liver failure with hepatic encephalopathy or bone marrow failure with fatal hemorrhages in various organs [5]. The only curative treatment of XLP is hematopoietic stem cell transplantation [6].

In our case, the HLH-2004 protocol, initiated on the 6^th^ hospitalization day, did not prevent the deterioration of the patient’s clinical status. Rituximab therapy has been reported to successfully induce remission in some cases of XLP [7,8]. Unfortunately, our patient was lost before we could start rituximab therapy.

Establishment of the genetic diagnosis in male children suspected to have XLP will enable valuable genetic counseling.

## Figures and Tables

**Table 1:**
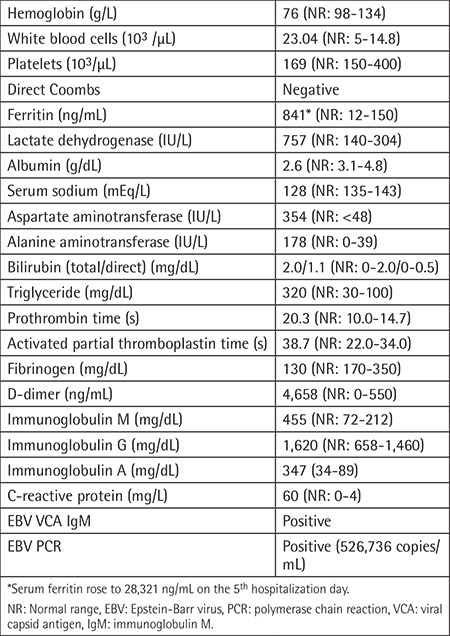
Laboratory findings of the patient.
